# Bacterial Filamentation Drives Colony Chirality

**DOI:** 10.1128/mBio.01542-21

**Published:** 2021-11-02

**Authors:** Andrés Aranda-Díaz, Cecilia Rodrigues, Alexandra Grote, Jiawei Sun, Carl Schreck, Oskar Hallatschek, Anton Souslov, Wolfram Möbius, Kerwyn Casey Huang

**Affiliations:** a Department of Bioengineering, Stanford University, Stanford, California, USA; b Living Systems Institute, University of Exeter, Exeter, United Kingdom; c Physics and Astronomy, College of Engineering, Mathematics and Physical Sciences, University of Exeter, Exeter, United Kingdom; d Department of Physics, University of California at Berkeley, Berkeley, California, USA; e Department of Physics, University of Bath, Bath, United Kingdom; f Department of Microbiology and Immunology, Stanford University School of Medicine, Stanford, California, USA; g Chan Zuckerberg Biohub, San Francisco, California, USA; Fred Hutchinson Cancer Research Center

**Keywords:** A22, MreB, anaerobic growth, cell wall, cephalexin, peptidoglycan, temperature, twisting, chirality, colony growth

## Abstract

Chirality is ubiquitous in nature, with consequences at the cellular and tissue scales. As Escherichia coli colonies expand radially, an orthogonal component of growth creates a pinwheel-like pattern that can be revealed by fluorescent markers. To elucidate the mechanistic basis of this colony chirality, we investigated its link to left-handed, single-cell twisting during E. coli elongation. While chemical and genetic manipulation of cell width altered single-cell twisting handedness, colonies ceased to be chiral rather than switching handedness, and anaerobic growth altered colony chirality without affecting single-cell twisting. Chiral angle increased with increasing temperature even when growth rate decreased. Unifying these findings, we discovered that colony chirality was associated with the propensity for cell filamentation. Inhibition of cell division accentuated chirality under aerobic growth and generated chirality under anaerobic growth. Thus, regulation of cell division is intrinsically coupled to colony chirality, providing a mechanism for tuning macroscale spatial patterning.

## INTRODUCTION

An object is chiral if it is distinguishable from its mirror image. Chirality is prevalent throughout nature at all scales, and stereoisomers are often functionally distinct, from our left and right hands to l- and d-amino acids that are used for metabolism/translation and bacterial cell wall synthesis, respectively. Chirality is manifest in polymers that form helices, such as bacterial flagella and cytoskeletal filaments (1, 2). Chirality can also be an intrinsic property of individual cells; for instance, myosin in *Drosophila* can reverse handedness in cells, which feeds forward to affect organ handedness (3). Chirality drives the development of left-right asymmetry generation in organs of Drosophila melanogaster (4) and in the Caenorhabditis elegans embryo (5). Plants twist as they grow, and mutations in SPIRAL2 change that twist from left- to right-handed; this handedness reversal is coupled to a switch from anisotropic growth to isotropic growth (6). However, it is largely unknown how chirality at the tissue and organismal scales is linked to cellular and molecular properties. Here, we investigate the link between chirality and growth at the micron scale of individual bacterial cells and chirality at the millimeter scale of colonies, visible to the naked eye.

**FIG 1:**
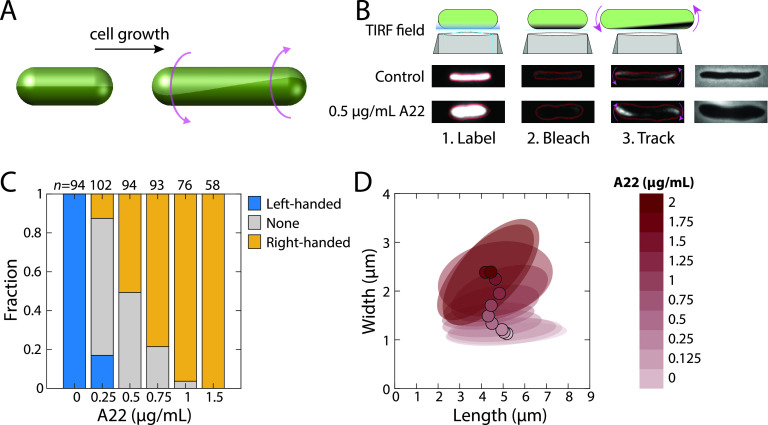
A22 reverses twisting handedness and alters cellular dimensions in E. coli DH5α. (A) Schematic of left-handed twisting during elongation of a rod-shaped cell. (B) In the Twist-n-TIRF method, the cell surface is labeled and the side of the cell closest to the coverslip is bleached using a TIRF microscope. During subsequent TIRF imaging at lower intensity, unbleached parts of the cell appear associated with cell twisting (see Materials and Methods). Phase-contrast images are displayed on the right. A22-treated E. coli DH5α cells are typically shorter and wider than untreated cells and twist with opposite handedness during the tracking step; note the appearance of fluorescence on the lower left and upper right in the A22-treated cell, as opposed to the upper left and lower right in the untreated cell. (C) In the absence of A22, virtually all E. coli DH5α cells exhibit left-handed twisting, while cells treated with 1.5 μg/ml A22 exhibit right-handed twisting. The number of cells (*n*) is indicated above each bar. (D) E. coli DH5α cell width during log-phase growth in liquid increases as a function of A22 concentration. For concentrations of <1 μg/ml, cell length decreases with increasing A22 concentration. Circles represent mean dimensions, and ellipses represent the covariance matrix of length and width. At least 50 cells were quantified for each condition.

Many rod-shaped bacterial cells exhibit twisting at the single-cell level during growth. In the Gram-negative bacterium Escherichia coli, growth occurs along the body of the cell and not at the poles (7). As the two ends move apart from one another, they also rotate in opposite directions, representing left-handed twisting (Fig. 1A) (8). Twisting has also been observed in Gram-positive Bacillus subtilis (9), in a right-handed manner (8). Bacterial growth requires expansion of the cell wall, a rigid macromolecule composed of cross-linked glycan strands (10) that is necessary and sufficient for cell shape determination (11). A biophysical model of cell wall growth quantitatively predicted the degree of cell twisting generated by a helical pattern of insertion, which produced cell wall material with the opposite types of handedness (8, 12). The bacterial actin homolog MreB (13), which is responsible for the spatiotemporal patterning of cell wall material (7), was required for cell twisting (8). The small molecule A22 depolymerizes MreB; at high concentrations, cells eventually round up and lyse (14). Twisting of E. coli cells is tunable: as cells widen under increasing sublethal levels of A22 treatment (15), the angle of MreB motion, which is thought to signify the placement of new strands of cell wall material (16), rotates and ultimately adopts an angle on the opposite side of the line perpendicular to the long axis of the cell, corresponding to a gradual conversion of twisting from left- to right-handed (15).

Bacterial colonies can also exhibit chirality during growth (17, 18). This effect is striking in experiments investigating range expansions, in which otherwise genotypically and phenotypically identical cells producing fluorescent proteins of two different colors (purely for the purposes of distinguishing genotypes) are inoculated onto an agar plate to grow into a colony. As the colony expands, cells on the exterior have preferential access to the uncolonized surface area and nutrients, leading to spatial segregation of the two fluorophores in well-defined “sectors” that expand outward, ultimately producing a pinwheel pattern. Boundaries between these sectors provide a frozen record of colony growth. On top of the wiggling motion of boundaries between sectors, the boundaries of bacterial species such as E. coli exhibit deterministic chirality, manifested as bending clockwise or counterclockwise, a consequence of nonradial movement along the edge of the colony (18). As with molluscan shells, where there is a deflecting force that remains perpendicular to the growing curves and causes bending (19), the pattern is well described by a logarithmic spiral, allowing quantification of the magnitude of colony chirality, or pitch, with the spiral’s chiral angle (θ; see Materials and Methods) (18). Ultimately, cells themselves are likely to generate the observed behavior at the colony scale, although colony chirality must also be dependent on environmental conditions, such as adhesion and surface wetness, both of which dictate movement of cells on the surface. Indeed, E. coli colony chirality was shown to be mediated by close interactions between cells and the surface, with the expression of pili and other adhesive structures suppressing chirality and agar stiffness affecting chirality (20). There are also strain-specific differences in colony chirality (20), even though twisting at the single-cell level is relatively constant (8). Modeling suggested that chirality can be an important population-level trait that mediates competition, invasion, and ultimately spatial structure within a bacterial community (50). Previous studies that attempted to explore the relationship between cell shape or cell wall synthesis and colony chirality made comparisons between different strains or species (20). However, a systematic interrogation of the links between single-cell properties and colony chirality through environmental, genetic, and physiological perturbations has not been undertaken. In particular, it is critical to employ a strategy that tunes behaviors such as twisting in a single organism in order to probe potential couplings with colony chirality.

Here, we set out to determine the relationship between cell shape, twisting, and handedness at the single-cell level and macroscopic colony chirality. Using single-cell and colony imaging, we found that A22 treatment and anaerobic growth inhibited growth and reduced colony chirality to near zero, making it unclear whether single-cell twisting was responsible for the change in chiral angle. Cells at the edge of the colony responded to A22 treatment by reducing their width and length. Chiral angle increased with increasing temperature, even at high temperatures that caused a decrease in growth, indicating that growth rate does not determine colony chirality. Across all conditions, the presence of chiral colonies was associated with filamentous cells at the edge of the colony, and antibiotic inhibition of division resulted in enhanced chirality. These results reveal a complex connection between single-cell dimensions and population-level spatial patterns, underscoring the role of cell division in determining colony chirality.

## RESULTS

### E. coli DH5α cells exhibit single-cell twisting similar to that of MG1655.

Chirality in E. coli colonies is readily observed in strain DH5α (17, 18), which has a clockwise rotation when viewed from the top. A recent comparison between E. coli strains revealed that on low-salt LB agar, E. coli MG1655 colonies also rotate clockwise but exhibit less pronounced chirality than DH5α colonies, possibly due to differences in extracellular structures and the ability to form biofilms that modify the interaction of cells with their substratum (20). To determine whether this difference in chirality may also be due to differences in twisting at the single-cell level (Fig. 1A), we utilized our previously developed Twist-n-TIRF method, where “TIRF” stands for total internal reflection fluorescence (15). In this method, cells are treated with the beta-lactam antibiotic cephalexin to block cell division, allowing for easier visualization of twisting. The cell wall is labeled uniformly with a stationary dye (in this case, wheat germ agglutinin [WGA] labeled with Alexa Fluor 488 [7]), and then the bottom of the cell is bleached by a TIRF laser. As the cell subsequently grows, twisting causes bright, unbleached regions to rotate into the TIRF imaging plane (Fig. 1B), and the handedness and degree of twisting can be computed from the direction and rate of fluorescence appearance, respectively (15). Virtually all DH5α cells clearly twisted in a left-handed manner on LB agarose pads (Fig. 1C), similar to the results of our previous measurements of MG1655 on EZ-Rich Defined Medium (EZ-RDM) pads (15) and consistent with the results of our previous study employing beads bound to the outer membrane (8). Thus, left-handed twisting is conserved between these E. coli strains and growth media.

Given the central role of MreB in single-cell twisting (8, 15), we next sought to quantify the effects of A22 treatment on DH5α. We treated log-phase DH5α cells expressing yellow or cyan fluorescent protein (YFP or CFP, respectively) from a plasmid for 3 h with a range of A22 concentrations from below to above the MIC (∼1 μg/ml) in LB (see Materials and Methods). At higher A22 concentrations, Twist-n-TIRF measurements revealed an increasing fraction of DH5α cells that exhibited right-handed or ambiguous twisting (Fig. 1C), similar to the handedness reversal of MG1655 (15). Cell shape also changed, as expected from previous experiments with MG1655 (15): DH5α cells increased in width and decreased in length as A22 concentration was increased (Fig. 1D and Fig. S1). Thus, across A22 concentrations, DH5α exhibits changes in twisting and cell shape similar to those of MG1655.

### A22 treatment reduces colony chirality.

**FIG 2:**
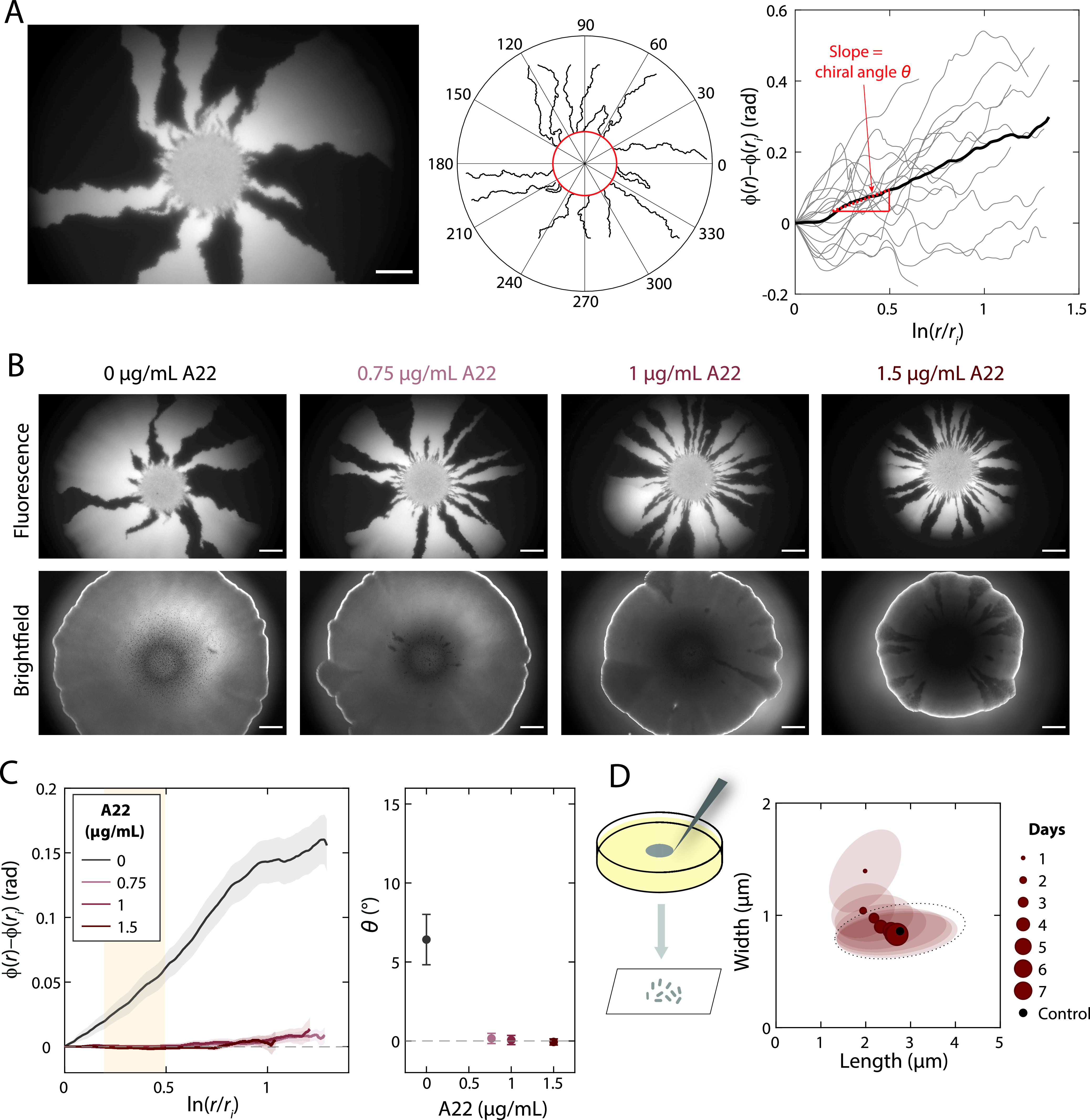
A22 treatment reduces colony chiral angle. (A, left) Genetic demixing during E. coli DH5α colony growth results in monoclonal sectors. Scale bar, 1 mm. (Middle) Although the shape of the boundaries between light (YFP) and dark sectors appeared stochastic, quantitative image analysis revealed an overall clockwise rotation of sector boundaries when the plate was viewed from the top (air interface). (Right) The slope (red) of the mean (thick black curve) is defined as the chiral angle. (B) Images of typical colonies after 7 days of growth on plates with various concentrations of A22 illustrate that colony growth is hindered by A22. The bright outline in the brightfield images denotes the colony border. Sector boundaries were straighter at higher A22 concentrations. Scale bars, 1 mm. (C, left) Mean rotation of sector boundaries at various A22 concentrations. (Right) The chiral angle decreases at higher A22 concentrations. Mean chiral angles were calculated for radii highlighted by the yellow region. Each data point is the average of results for ≥5 colonies. Error bars represent 1 standard deviation. (D) During colony growth in the presence of 1.5 μg/ml A22, cellular dimensions gradually revert back to those of cells grown in colonies in the absence of A22, suggesting morphological adaptation to A22.

The inversion of twisting handedness due to A22 treatment provides a qualitative change in cellular behavior through which to interrogate the connection between single-cell and colony-level behaviors. If single-cell twisting is the determinant of colony chirality, we should see a change from clockwise to counterclockwise rotation within colonies as single-cell twisting changes with increasing A22 concentration. To test this hypothesis, we grew mixed colonies of YFP- and CFP-expressing DH5α on LB plates at 37°C with a range of A22 concentrations and imaged the colonies after 7 days of growth. As expected, the frozen record revealed that colonies quickly developed into sectors of single colors (Fig. 2A). We segmented the images, identified sector boundaries, plotted the change in angle against a function of colony radius, and computed the chiral angle (θ) (Fig. 2A; see also Materials and Methods). In the absence of A22, the chiral angle was 6.4° (Fig. 2B and C), similar to previous measurements (17, 20). A22 treatment reduced the chiral angle to approximately zero (Fig. 2B and C), but even at high concentrations, there was no clear evidence of a reversal of handedness, in seeming contradiction to our hypothesis.

In a recent study, we showed that E. coli adapts to the cell-widening effects of A22 treatment by increasing the expression of *mreB*, resulting in a subsequent decrease in cell width (22). If cellular dimensions changed over the course of colony growth, such changes may have confounded our above test of the effect of A22 and single-cell twisting on colony chirality. To test whether cells on LB plates with A22 changed shape over time, we sampled cells from the edge of colonies once per day and imaged the cells to quantify their dimensions (see Materials and Methods). As we suspected, cell width decreased steadily over the course of a week, reaching a relatively constant width displayed by cells on plates without A22 (Fig. 2D); coincident with the width decrease, the length gradually increased (Fig. 2D). For the widest cells (∼1.4 μm), the cell width measured on day 1 on LB plus 1.5 μg/ml A22 was equivalent to the cell width in liquid LB plus 0.5 μg/ml A22 (Fig. 1D). Under this condition, ∼60% of cells exhibited no or ambiguous twisting (Fig. 1C), consistent with a lack of chirality at the colony level. Thus, the dynamics of cell shape on plates prevent a direct test of whether A22-mediated reversal of single-cell twisting handedness necessarily reverses colony chirality handedness.

### Heterologous expression of a foreign cell wall synthesis enzyme reverses single-cell twisting, but colonies do not exhibit chirality.

**FIG 3:**
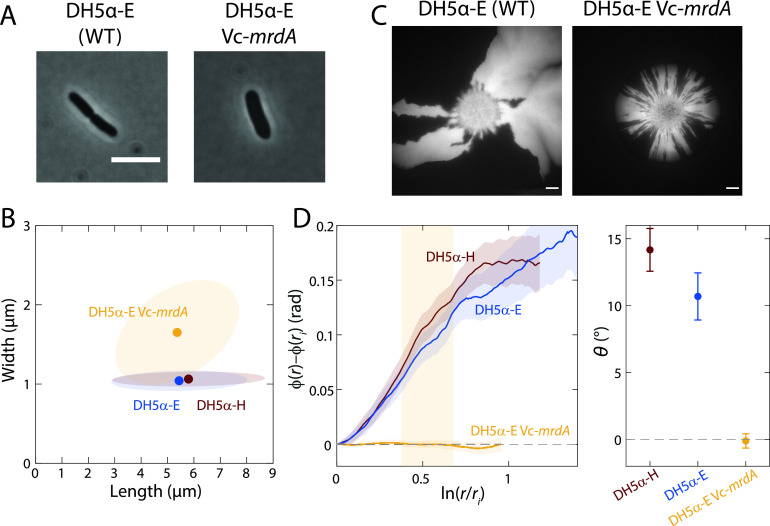
A genetic perturbation that causes cells to become wider eliminates colony chirality. (A, B) Heterologous expression of the *mrdA* gene from Vibrio cholerae (Vc-*mrdA*) in the E. coli DH5α-E Δ*mrdA* background increases cell width during log phase in liquid growth relative to that of wild-type (WT) DH5α-H or DH5α-E cells. (B) Circles represent mean values, and ellipses represent the covariance of width and length. At least 50 cells were quantified for each strain. Scale bar, 5 μm. (C, D) Colony chirality is reduced by heterologous expression of Vc-*mrdA*. (C) Image of typical colonies after 7 days of growth. Scale bars, 1 mm. (D, left) Mean rotation of sector boundaries for each strain. (Right) The chiral angle is essentially zero for the Vc-*mrdA* strain. Mean chiral angles were calculated for radii highlighted in the yellow region. Each data point is the average of results for ≥5 colonies. Error bars represent 1 standard deviation.

To circumvent the recovery of cellular dimensions and the potential consequences on twisting associated with A22 treatment, we sought an alternate mechanism for altering single-cell twisting. We previously showed that heterologous expression of *mrdA* from Vibrio cholerae (Vc-*mrdA*), which encodes the essential transpeptidase PBP2 (23), caused E. coli MG1655 cells lacking the endogenous *mrdA* gene to widen and to reverse twisting handedness, similarly to A22 treatment (15). We constructed Δ*mrdA* strains with constitutive expression of Vc-*mrdA* and a plasmid coding for CFP or YFP, resulting in DH5α-E-CFP Vc-*mrdA* and DH5α-E-YFP Vc-*mrdA* (see Materials and Methods). The mean cell width of these strains was significantly larger than for the corresponding control, DH5α-E, or for the DH5α-H strain used in the experiments described above (Fig. 3A and B). DH5α-E Δ*mrdA* Vc-*mrdA* cells were highly sensitive to cephalexin, resulting in rapid lysis during Twist-n-TIRF experiments that precluded measurement of single-cell twisting. Unlike A22-treated wild-type cells, untreated fluorescent DH5α-E Δ*mrdA* Vc-*mrdA* cells remained wider and shorter than wild-type cells over 5 days of growth in colonies (Fig. S2). Colonies displayed highly reduced chiral angles (Fig. 3C and D), leaving it unclear whether reversal of handedness at the single-cell level results in reversed handedness of colony chirality.

10.1128/mBio.01542-21.2 FIG S1 Fluorescent-protein-expressing strains have similar responses to A22 treatment. During liquid growth, the cell widths of E. coli DH5α-H expressing CFP and YFP, measured during log-phase growth in liquid, increased as the A22 concentration increased. Cell length decreased with increasing A22 concentrations up to 1 μg/ml. Circles represent mean dimensions, and ellipses represent the covariance matrix of length and width. At least 50 cells were quantified for each condition. Download FIG S1, PDF file, 0.1 MB. Copyright © 2021 Aranda-Díaz et al. 2021 Aranda-Díaz et al. https://creativecommons.org/licenses/by/4.0/ This content is distributed under the terms of the Creative Commons Attribution 4.0 International license.

10.1128/mBio.01542-21.3 FIG S2 E. coli DH5α-E Δ*mrdA* cells with heterologous expression of the *mrdA* gene from Vibrio cholerae (Vc-*mrdA*) remain wider than wild-type DH5α-H or DH5α-E cells after 5 days of colony growth. Circles represent mean values, and ellipses represent the covariance of width and length. At least 50 cells were quantified for each strain. Download FIG S2, PDF file, 0.08 MB. Copyright © 2021 Aranda-Díaz et al. 2021 Aranda-Díaz et al. https://creativecommons.org/licenses/by/4.0/ This content is distributed under the terms of the Creative Commons Attribution 4.0 International license.

### Quenching of colony chirality between two surfaces is likely due to a lack of twisting in anaerobic environments.

**FIG 4:**
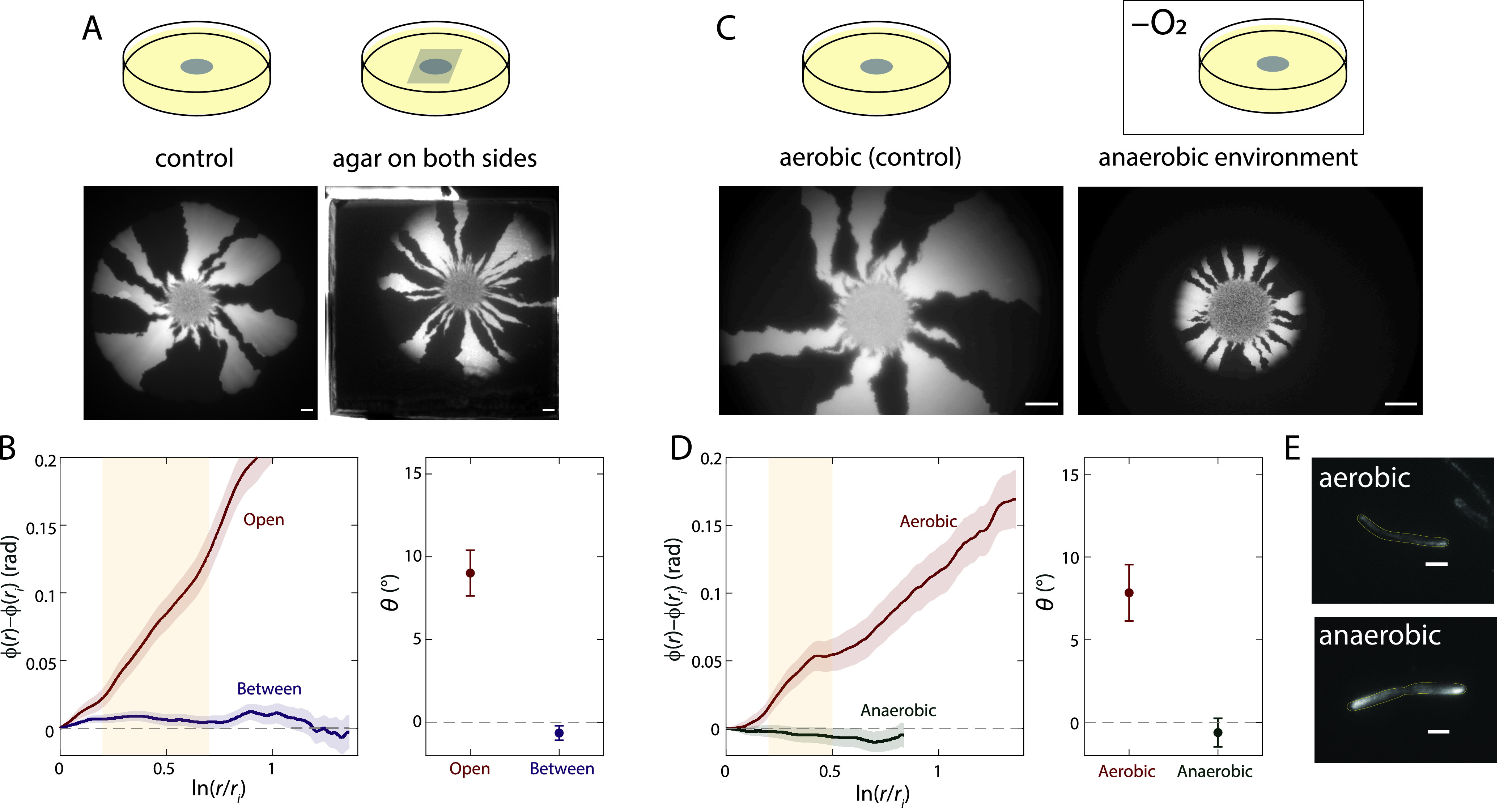
Colony chirality is decreased during growth when sandwiched between agar surfaces or under anaerobic conditions. (A, top) Schematic of control experiments with an air-agar interface (“Open,” left) and sandwiched between two agar surfaces (“Between,” right) (see Materials and Methods). (Bottom) Representative colonies for each condition. Sector boundaries were straighter in the sandwiched colony. Scale bars, 1 mm. (B, left) Mean rotation of sector boundaries under each condition in panel A. (Right) The chiral angle is essentially zero for sandwiched colonies. Mean chiral angles were calculated for radii highlighted in the yellow region. Each data point is the average of results for ≥5 colonies. Error bars represent 1 standard deviation. (C, top) Schematic of growth under aerobic conditions and in an anaerobic chamber. (Bottom) Representative colonies for each condition. Sector boundaries were straighter during anaerobic growth. Scale bars, 1 mm. (D, left) Mean rotation of sector boundaries under each condition in panel C. (Right) The chiral angle is essentially zero during anaerobic growth. Mean chiral angles were calculated for radii highlighted in the yellow region. Each data point is the average of results for ≥5 colonies. Error bars represent 1 standard deviation. (E) DH5α cells still exhibit twisting at the single-cell level during anaerobic growth at 37°C, as revealed by Twist-n-TIRF. For both aerobic and anaerobic growth, all cells whose handedness could be reliably classified were left-handed (*n *= 43, aerobic; *n *= 31, anaerobic). Scale bars, 2 μm.

We hypothesized that colonies sandwiched between two agar surfaces should not exhibit any chirality based on symmetry considerations, independent of changes in cell shape. To test this hypothesis, we inoculated a droplet of CFP-expressing and YFP-expressing DH5α cells as before, allowed the droplet to dry, and then placed another large agar pad on top of the agar plate (Fig. 4A). The colony continued to expand between these two surfaces, presumably due to the lack of agar cross-linking between the two pads compared to that within the pads. Cells emitted very little fluorescence, which we surmised was due to the depletion of oxygen as the colony grew; anaerobic conditions prevent maturation of fluorescent proteins (24). GFP matures more readily at low temperatures (25); hence, we incubated sandwiched colonies at 4°C (see Materials and Methods) and then quantified the fluorescence patterns. The sector boundaries were essentially achiral (Fig. 4A and B; Fig. S3).

10.1128/mBio.01542-21.4 FIG S3 Colony chirality is reduced when grown sandwiched between two agar surfaces. Shown is a biological replicate of the experiment in Fig. 4B. (Left) Mean rotation of sector boundaries in the open or sandwiched configuration. (Right) The chiral angle was zero or slightly negative for sandwiched colonies. Each data point is the average of results for ≥5 colonies. Error bars represent 1 standard deviation. Download FIG S3, PDF file, 0.2 MB. Copyright © 2021 Aranda-Díaz et al. 2021 Aranda-Díaz et al. https://creativecommons.org/licenses/by/4.0/ This content is distributed under the terms of the Creative Commons Attribution 4.0 International license.

To test whether the absence of chirality was due to the restoration of symmetry at the interface or to the depletion of oxygen, we grew a mixed colony on a single agar surface under anaerobic conditions. We observed little to no chirality (Fig. 4C and D), indicating that anaerobic growth is sufficient to abolish colony chirality. Colonies were smaller after 7 days of anaerobic growth than they were after aerobic growth. To test whether anaerobic growth abolished single-cell twisting, we performed Twist-n-TIRF under anaerobic conditions (see Materials and Methods). Cells continued to twist in a left-handed fashion (Fig. 4E); thus, lack of colony chirality under anaerobic conditions is not due to the lack of single-cell twisting. Instead, these findings suggest that another, oxygen-dependent factor influenced the level of colony chirality.

### Temperature alters growth rate and chirality without changing cell twisting.

**FIG 5:**
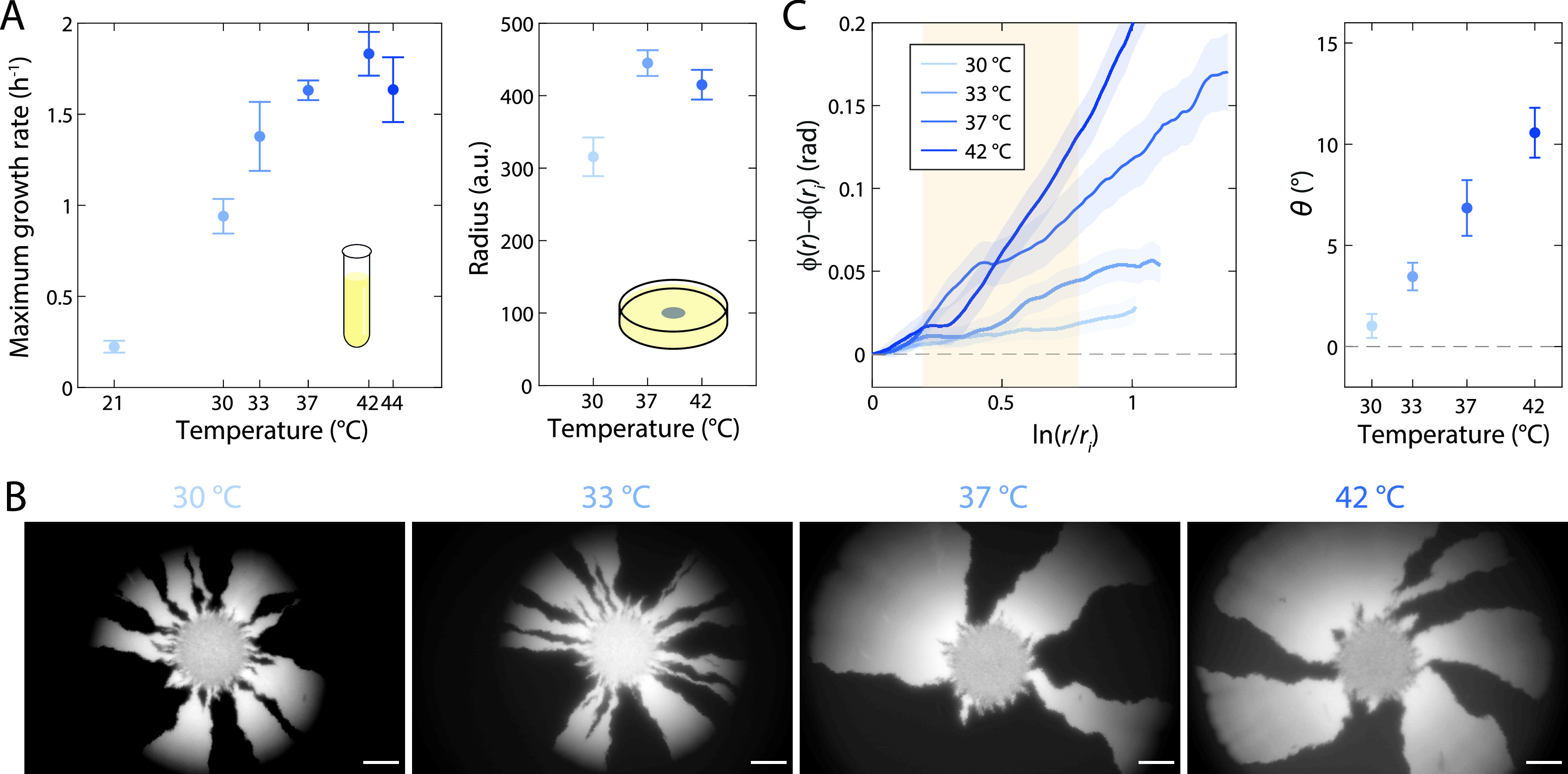
Chiral angle increases with increasing temperature. (A) E. coli DH5α growth depends on temperature. (Left) The maximum growth rate in liquid peaks at 42°C. (Right) Colony radius is higher at 37°C than at 30 or 42°C. a.u., arbitrary units. (B) Images of representative colonies show increasing chirality at higher temperatures. Scale bars, 1 mm. (C, left) Mean rotation of sector boundaries at each temperature. (Right) The chiral angle continues to increase with increasing temperature, even at 42°C. Mean chiral angles were calculated for radii highlighted in the yellow region. Each data point is the average of results for ≥5 colonies. Error bars represent 1 standard deviation.

After 7 days, colonies grown on LB plus A22 (Fig. 2B) or anaerobically (Fig. 4C) were significantly smaller than when they were grown aerobically on LB alone (Fig. 2A and B and 4C). Thus, we sought to test whether growth rate was a major determinant of colony chirality. Temperature is well known to modulate growth rate (26). In liquid, the maximum growth rate of DH5α increased as a function of temperature up to an optimal temperature of ∼42°C (Fig. 5A; Fig. S4). We grew mixed colonies across a range of temperatures and imaged them after 7 days (Fig. 5B). Colony size was also temperature dependent, with smaller colonies at 30 and 42°C than at 37°C (Fig. 5A). However, chiral angle increased monotonically with temperature up to 42°C (Fig. 5C), indicating that chirality is regulated by a factor other than growth rate that changes with temperature.

10.1128/mBio.01542-21.5 FIG S4 Growth curves of E. coli DH5α grown in liquid at various temperatures. The slope of the dashed lines defined the maximum growth rate in Fig. 5A. Each plot shows 20 replicate growth curves. Download FIG S4, PDF file, 0.2 MB. Copyright © 2021 Aranda-Díaz et al. 2021 Aranda-Díaz et al. https://creativecommons.org/licenses/by/4.0/ This content is distributed under the terms of the Creative Commons Attribution 4.0 International license.

### Filamentation is linked to the extent of colony chirality.

**FIG 6:**
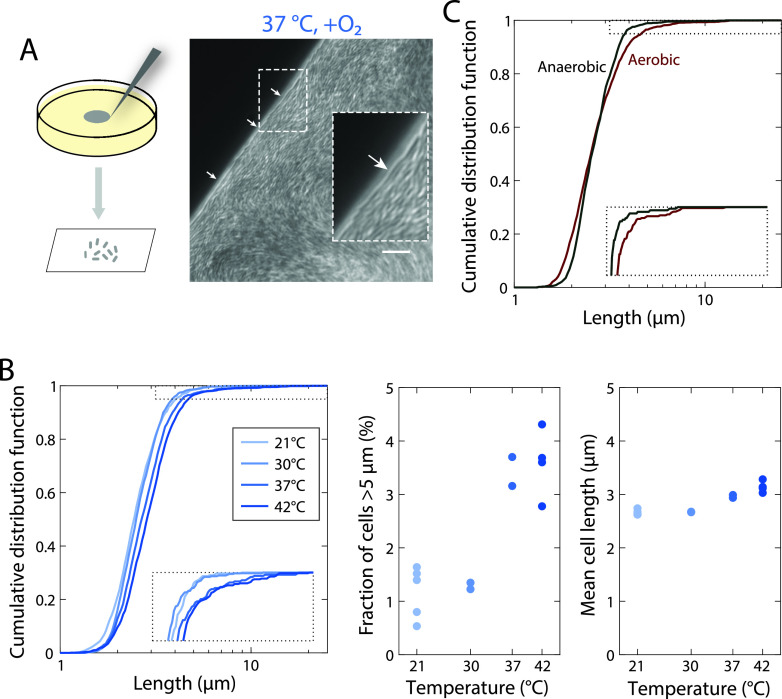
Conditions with enhanced colony chirality exhibit increased fractions of filamentous cells at the colony edge. (A, left) Schematic of sampling from the colony edge. (Right) Imaging of the colony edge reveals filamentous cells at the border (arrows). The inset is a 200% magnification of the region surrounded by the dashed white line, highlighting a filamentous cell. (B) At higher temperatures, a larger fraction of the population exhibits filamentation. (Left) The cumulative distribution function of cell length shifted to the right at increasing temperatures, and very long, filamentous cells were found at high temperatures. The inset is a magnification of the dotted region. Pairwise differences of the distributions are all significant based on Kolmogorov-Smirnov (*P < *0.002) and Mann-Whitney (*P < *0.004) tests. (Middle) The fraction of cells with a length of >5 μm in samples from the edge of colonies increased at increasing temperature. (Right) The mean cell length also increased slightly with increasing temperature. Each circle was computed from ≥16 fields of view from a sample from a distinct colony. (C) During aerobic growth, a larger fraction of the population exhibits filamentation than anaerobic growth. The inset is a magnification of the dotted region. The difference between cumulative distribution functions is significant based on a Kolmogorov-Smirnov test (*P = *10^−5^), not on a Mann-Whitney test (*P = *0.13).

Single-cell twisting was approximately constant across temperatures (Fig. S5), again highlighting that another factor must dictate chirality. Motivated by our observations that A22 treatment reduced the cell aspect ratio (Fig. 1D) and chiral angle (Fig. 2C), we measured log-phase DH5α cellular dimensions in liquid culture at various temperatures. Length and width generally increased with temperature (27), with cells at 42°C more than twice as long as those at 21°C (Fig. S6). To verify whether cells were similarly elongated in colonies *in situ*, we imaged colonies directly and identified filamentous cells at the extreme edge of colonies with pronounced chirality (37°C and 42°C) (Fig. 6A and B), while less filamentation was apparent in colonies with little chirality (lower temperatures and anerobic growth) (Fig. 6; Fig. S7). Because of the difficulty in segmenting individual cells from *in situ* images of the colony, we sampled and imaged cells from the colony edge. We surmised that higher cell density in images was indicative of more sampling toward the center of the colony. Thus, to correct for variability in sampling location, we focused our analysis on sets of images with similar cell densities (Fig. S8). As in liquid, mean cell length increased with temperature, and colonies grown at higher temperatures displayed more filamentous cells (Fig. 6B). Interestingly, cells grown in liquid had similar cellular dimensions aerobically and anaerobically, but cells sampled from the edge of colonies grown anaerobically were wider (Fig. S9A) and exhibited less filamentation (Fig. 6C) than those from the edge of aerobic colonies. These observations are consistent with the hypothesis that some degree of cell filamentation is necessary for colony chirality.

10.1128/mBio.01542-21.6 FIG S5 Twisting rates are similar across temperatures. Fluorescence recovery (middle) after photobleaching of the bottom surface during Twist-n-TIRF experiments (left) was used to estimate the twisting rate of 12 cells at each temperature (right) (see Materials and Methods). Scale bars, 2 μm. All cells whose handedness could be reliably classified were left-handed (*n *= 67, 30°C; *n *= 43, 37°C). Download FIG S5, PDF file, 1.4 MB. Copyright © 2021 Aranda-Díaz et al. 2021 Aranda-Díaz et al. https://creativecommons.org/licenses/by/4.0/ This content is distributed under the terms of the Creative Commons Attribution 4.0 International license.

10.1128/mBio.01542-21.7 FIG S6 Cell length generally increases with increasing temperature. Cell length measured during log-phase growth in liquid increased as temperature increased up to 42°C. Circles represent mean dimensions, and ellipses represent the covariance matrix of length and width. At least 50 cells were quantified for each condition. Download FIG S6, PDF file, 0.08 MB. Copyright © 2021 Aranda-Díaz et al. 2021 Aranda-Díaz et al. https://creativecommons.org/licenses/by/4.0/ This content is distributed under the terms of the Creative Commons Attribution 4.0 International license.

10.1128/mBio.01542-21.8 FIG S7 Colonies grown at high temperatures have more filamentous cells at the border than colonies grown at low temperatures or under anaerobic conditions (–O_2_). (Insets) Magnifications (160%) of regions highlighted by the dashed white line. Arrows denote filamentous cells. Scale bars, 10 μm. Download FIG S7, TIF file, 2.5 MB. Copyright © 2021 Aranda-Díaz et al. 2021 Aranda-Díaz et al. https://creativecommons.org/licenses/by/4.0/ This content is distributed under the terms of the Creative Commons Attribution 4.0 International license.

10.1128/mBio.01542-21.9 FIG S8 Cell length varies with cell density. Cells were sampled from the edge of colonies grown at 37 or 42°C. For samples with a higher cell density (based on the number of cells in the field of view, likely due to being sampled farther from the colony edge), the fraction of cells longer than 5 μm (left) and the mean cell length (right) were lower. Each circle was computed from ≥16 fields of view from a sample from a distinct colony. Download FIG S8, PDF file, 0.07 MB. Copyright © 2021 Aranda-Díaz et al. 2021 Aranda-Díaz et al. https://creativecommons.org/licenses/by/4.0/ This content is distributed under the terms of the Creative Commons Attribution 4.0 International license.

**FIG 7:**
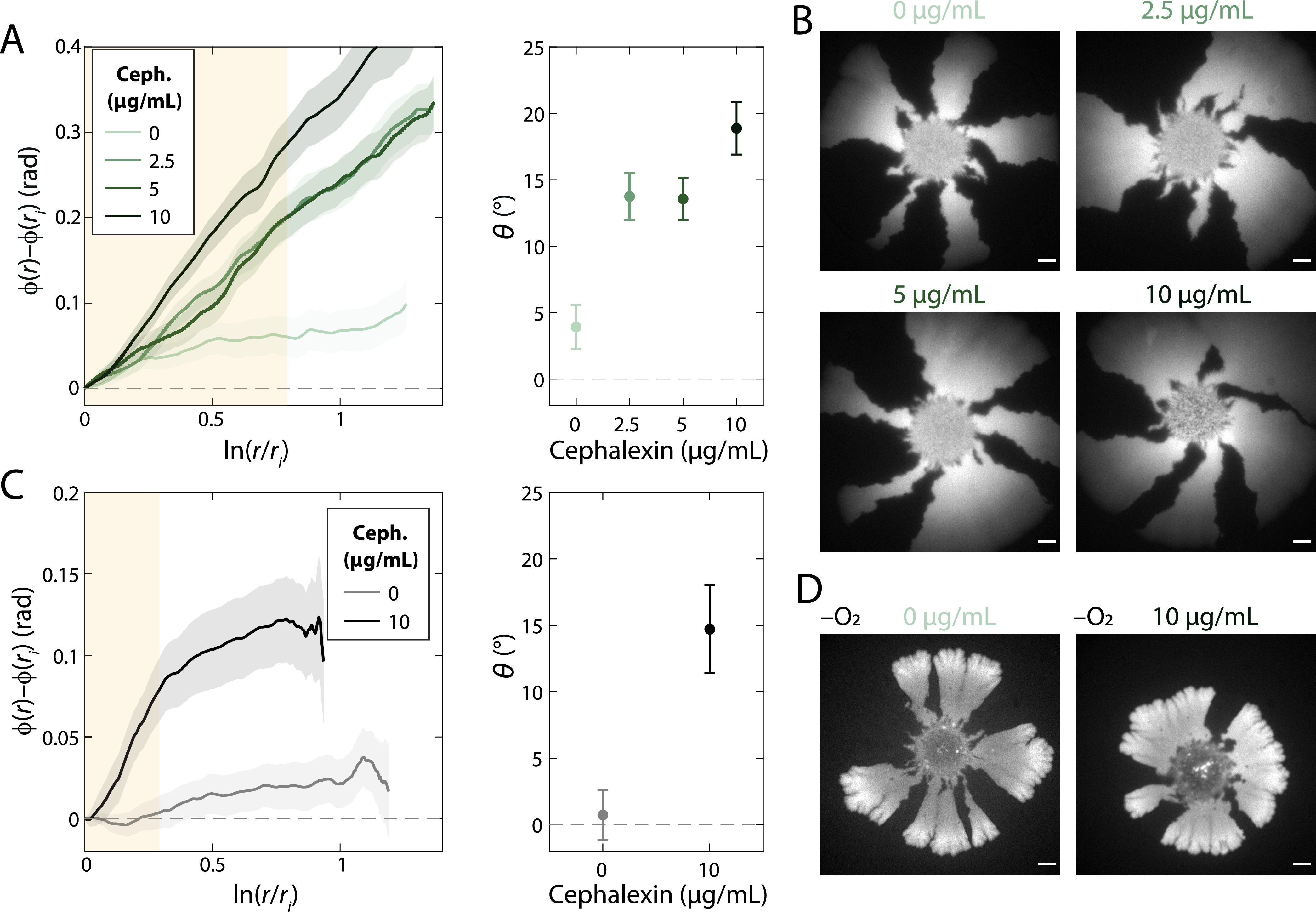
Division inhibition using cephalexin results in enhanced chirality during aerobic growth and the introduction of chirality during anaerobic growth. (A, left) Mean rotation of sector boundaries of aerobically grown wild-type DH5α colonies with various concentrations of cephalexin. (Right) The chiral angle increases with increasing cephalexin concentration. Mean chiral angles were calculated for radii highlighted in the yellow region. Each data point is the average of results for ≥5 colonies. Error bars represent 1 standard deviation. (B) Representative colonies grown aerobically with various concentrations of cephalexin. Scale bars, 1 mm. (C, left) Mean rotation of sector boundaries of anaerobically grown wild-type DH5α colonies without and with cephalexin. (Right) In the presence, but not in the absence, of cephalexin, colonies exhibit chirality. Mean chiral angles were calculated for radii highlighted in the yellow region. Each data point is the average of results for ≥5 colonies. Error bars represent 1 standard deviation. (D) Representative colonies grown anaerobically without and with 10 μg/ml cephalexin. Scale bars, 1 mm.

10.1128/mBio.01542-21.10 FIG S9 Cells in anaerobically grown colonies are wider than in aerobically grown colonies, and cephalexin treatment increases colony chirality. (A) Cells were sampled from the edge of colonies. Circles represent mean dimensions, and ellipses represent the covariance matrix of length and width. At least 50 cells were quantified for each condition. (B) Shown is a biological replicate of the experiment in Fig. 7A. (Left) Mean rotation of sector boundaries of aerobically grown wild-type DH5α colonies with various concentrations of cephalexin. (Right) The chiral angle increases with increasing cephalexin concentration. Each data point is the average of results for *≥*5 colonies. Error bars represent 1 standard deviation. Download FIG S9, PDF file, 0.5 MB. Copyright © 2021 Aranda-Díaz et al. 2021 Aranda-Díaz et al. https://creativecommons.org/licenses/by/4.0/ This content is distributed under the terms of the Creative Commons Attribution 4.0 International license.

### Inhibition of cell division enhances chirality.

To determine whether filamentation and chirality are causally linked, we sought to increase the fraction of filamentous cells within a colony. We grew mixed cultures at 37°C on various concentrations of cephalexin, a beta-lactam antibiotic that inhibits the division-specific transpeptidase PBP3 (28). Another division-inhibiting beta-lactam, aztreonam, led to rapid selection of resistant mutants and thus was not useful for this study. At higher cephalexin concentrations, colonies exhibited a pronounced chirality similar to that at 42°C, and chiral angle increased in a dose-dependent manner (Fig. 7A and B; Fig. S9B). To test whether cephalexin would introduce chirality in situations where the chiral angle was close to zero, we grew mixed cultures anaerobically on LB plus 10 μg/ml cephalexin plates. Remarkably, cephalexin-treated colonies exhibited obvious chirality (Fig. 7C and D). Thus, inducing filamentation is sufficient to introduce or accentuate colony chirality.

## DISCUSSION

In this study, we systematically measured the degree and handedness of twisting at the single-cell level across many perturbations and determined that while changes that perturb single-cell twisting alter colony chirality, changes that perturb colony chirality do not necessarily alter single-cell twisting. It is clear that many factors interact to determine colony structure, including forces between cells and the accessibility of nutrients (29), cellular geometry (30), and interactions of cells with each other and with the surface (20). Previous findings connecting cell surface appendages with colony chirality (20), which implied that surface attachment plays a role in generating chirality, are not inconsistent with those of our study, since the dependence of agar properties and surface attachment on temperature, cell filamentation, and oxygen are unknown. Our colony chirality observations are phenomena that are either intrinsically three-dimensional or originate from cells being subjected to a solid surface on one side and an air surface on the other, as confining the cells between two agar pads may eliminate chirality by removing the air-agar asymmetry (Fig. 4A). Regardless, our data highlight the role of cell filamentation in establishing colony chirality, with division-inhibiting cephalexin treatment leading to the introduction of chirality in colonies grown anaerobically (Fig. 7). Moreover, filamentation is the variable that unifies colony chirality across all of our growth conditions. These results reveal a complex connection between single-cell dimensions and population-level spatial patterns (31, 32).

Our original motivation for this study was to establish whether colony chirality can be traced back to single-cell behaviors. While we have shown that cell filamentation is coupled to the generation of colony chirality, it remains unclear whether single-cell twisting is also a factor. A22 treatment does reduce chirality (Fig. 2), as does genetic manipulation of the cell wall synthesis machinery (Fig. 3). Moreover, single-cell twisting and colony chirality each have a defined handedness in the absence of A22. However, it remains unclear whether and how handedness at the microscopic scale determines handedness at macroscopic scales.

The connection between filamentation, and more generally cell shape, and colony patterning was unexpected, in part because the regulation of division across environmental conditions, such as A22 treatment, anaerobic growth, and temperature, are poorly understood. In the case of A22, it was critical to measure cell shape at the edge of the colony, which revealed that the cell width phenotype characteristic of A22 treatment on short time scales was reversed after several days (Fig. 2D), likely due to transcriptional adaptation (22). It is intriguing that regulation of cell division takes place on such long time scales. The tunability of chirality using cephalexin provides an interesting control knob for the design of macroscale patterns in bacterial communities.

What are the origins of colony chirality? Ultimately, chirality must materialize from some molecular symmetry breaking, and while the cell wall is an enticing candidate, the extracellular environment cannot be ruled out. This knowledge gap motivates the quantification of single-cell twisting and colony chirality across more strains and species to determine whether the two are generally coupled. An ideal demonstration would be the reversal of handedness of a single species, as we attempted in this work, although it may be impossible to construct a right-handed E. coli cell that is stable during filamentation without reprogramming many cell wall synthesis components. Comparisons with B. subtilis, which displays right-handed twisting during single-cell growth (8, 9), may provide interesting comparisons to E. coli in future investigations, particularly due to its higher mechanical flexibility (33).

A wealth of exotic patterns and mechanical properties can emerge from chiral constituents exerting forces on each other and their environments (34–36), including periodic crystals of rotating particles (37–39) that can synchronize into exotic phases (40). Mixtures of oppositely rotating particles tend to phase separate (41, 42), leading to complex structures and flows at chiral-phase interfaces (43, 44). Analyzing flow patterns and excitations of chiral active fluids has led to the design of topological states of matter (45, 46) and predictions of novel hydrodynamic responses (47, 48) that have been experimentally measured (49). Biological settings, such as colony chirality, may provide even more complex ways to connect microscopic forces of chirally active components to macroscopic handedness. For example, the observation that filamentation in only a small percentage of cells is sufficient to induce chirality in anaerobically grown colonies motivates the study of mixtures of active particles with a heterogeneous distribution of geometries. The longer cells may induce longer spatial correlation lengths of cellular alignment, and they may create barriers that corral cells to one side of the sector boundary, both of which would reinforce a small chiral bias. Imaging is challenging away from the colony edge due to the three-dimensional nature of colony growth; hence, it remains unclear whether the presence of filamentous cells is important only at the boundary or throughout the colony.

From an ecological and evolutionary perspective, what is the relevance of colony chirality? Building on recent work suggesting that chirality is an important ecological trait (50), our work suggests that colony-scale patterning has likely applied selective pressures on division at the cellular scale. Similarly, to design the spatial structure of bacterial communities, it is advantageous to control the emergent pattern of cell growth rather than to have to establish patterns from the start. To achieve this control will ultimately require a mechanistic model for colony chirality, the development of which will be facilitated by the discovery that cellular properties, such as cell length, are critical parameters.

## MATERIALS AND METHODS

### Bacterial strains and plasmids.

Strains used in this study are described in Table S1 in the supplemental material. In brief, we used three pairs of strains. In each pair, the two strains have a common genetic background and carry plasmids expressing CFP or Venus YFP. The pair used for the majority of experiments (DH5α-H-CFP and DH5α-H-YFP) is identical to the one used in previous studies reporting colony chirality (17, 18, 20). In each pair, there were no obvious fitness differences between the two strains (both strains were able to form sectors).

10.1128/mBio.01542-21.1 TABLE S1 Bacterial strains used in this study. Download Table S1, PDF file, 0.03 MB. Copyright © 2021 Aranda-Díaz et al. 2021 Aranda-Díaz et al. https://creativecommons.org/licenses/by/4.0/ This content is distributed under the terms of the Creative Commons Attribution 4.0 International license.

For this study, we constructed E. coli DH5α Δ*mrdA* strains with constitutive expression of V. cholerae *mrdA* (Vc-*mrdA*) by replacing the genomic E. coli *mrdA* (Ec-*mrdA*) sequence with a 4,169-bp PCR fragment encoding the Vc-*mrdA* sequence and the kanamycin resistance cassette, followed by selection for stability. This procedure resulted in strains DH5α-E-CFP Vc-*mrdA* and DH5α-E-YFP Vc-*mrdA*. Control experiments required the use of strains derived from the corresponding ancestor, DH5α-E-CFP or -YFP.

Construction of the DH5α Δ*mrdA* strain was as follows. The Vc-*mrdA* DNA fragment was amplified from the pww308-VcmrdA construct (15) with primers that have 20 nucleotides of homology to the plasmid DNA sequence and 50 nucleotides of homology to the genomic Ec-*mrdA* target sequence, specifically delmrdAFor (5′-ACGCAGCGGATGAAACTACAGAACTCTTTTCGCGACTATACGGCAGAGCCACGTTGTGTCTCAAAATC-3′) and delmrdARev (5′-TATCCGTCATGATTAATGGTCCTCCGCTGCGGCAACCGCTGGATTTTCCGCATCCTAGGTCATGGCTGTATTAC-3′). The DH5α strain lacks the ability to homologously recombine. Therefore, E. coli strain DH5α (Invitrogen) was first transformed with the pSIM5 plasmid (pSC101 *repA*^ts^), encoding Red recombination proteins (51). Selection of the transformed cells was performed by growing the cells in Lennox LB in the presence of 17.5 μg/ml chloramphenicol at 30°C to allow pSIM5 replication, producing strain eCR106, which was able to perform homologous recombination. eCR106 cells were transformed with the gel-purified Vc-*mrdA* DNA fragment via electroporation as follows. eCR106 cells were grown at 30°C to optical densities (ODs) of 0.4 to 0.5, transferred to 42°C for 15 min for induction of Red proteins, and then transferred to ice for 10 min. Cells were centrifuged at 3,000 × *g*, and the pellet was washed with ice-cold water twice before resuspension of the cells in 10% glycerol solution for electroporation using a Gene Pulser XCell (Bio-Rad) electroporator with the preset bacterial protocol for E. coli using a 1-mm cuvette. After transformation, cells were incubated at 30°C for 2 h before selection on Lennox LB agar plates with 25 μg/ml kanamycin. Transformed cells were identified by colony PCR with the primers delBFor (5′-ACATCATCGCCTTAGACGTTC-3′) and delBRev (5′-AGATGGACTTTATCCCAGAATG-3′) for the upstream/downstream E. coli *mrdA* sequence and primers delCFor (5′-AGCGGATGAAACTACAGAACTC-3′) and delCRev (5′-CGGCAACCGCTGGATTTTC-3′) for the insert. Colony PCR identified fragments of two sizes (approximately 4,000 and 5,000 bp); we selected for the shorter fragment, which appeared more stable and, based on sequencing of the amplicon, corresponded to a deletion in the promoter.

Colonies were picked and grown in Lennox LB at 37°C to remove the pSIM5 plasmid, creating strain eCR111. pSIM5 loss was confirmed by the absence of growth under chloramphenicol selection.

CFP/YFP plasmids for DH5α-E-CFP Vc-*mrdA* and -YFP Vc-*mrdA* and DH5α-E-CFP and -YFP were constructed by cloning the fluorescent protein coding sequence from strains DH5α-H-CFP and -YFP into pSTV28 (TaKaRa Bio Europe SAS) with EcoRI/SalI restriction sites. Cells were transformed with CFP/YFP constructs via electroporation.

### Growth of mixed colonies.

A step-by-step protocol is available online on protocols.io (https://doi.org/10.17504/protocols.io.bx3epqje). In short, agar plates (6 cm in diameter) were prepared with 10 ml of Lennox LB (RPI catalog no. L24066; Melford catalog no. L24060 for colonies sandwiched between agar slabs and corresponding control experiments) with 1.5% agar (BD catalog no. 214530) and left on the bench overnight. Plates were used the next day or stored at 4°C. As necessary, A22 or cephalexin was added from frozen stocks to the liquid after autoclaving the liquid. To initiate colony growth, the appropriate pair of fluorescent E. coli strains was grown overnight at 37°C in liquid Lennox LB. Both cultures were diluted 10-fold in fresh LB and mixed. One-microliter droplets were pipetted onto prewarmed plates and grown at 37°C in a heated room inside plastic containers with wet paper towels and beakers with water to conserve humidity, typically for 7 to 8 days. Anaerobic growth experiments were carried out in a custom anaerobic chamber (Coy Lab Products). Colony growth between agar surfaces was achieved by cutting an agar pad out of a fresh plate and placing it upside down onto freshly inoculated cultures.

### Sampling from mixed colonies.

Cells were sampled from the border of colonies using a 20-μl LTS micropipette tip (Rainin). After touching the edge of the colony, cells were resuspended in 20 μl phosphate-buffered saline (PBS), and 1 μl was spotted onto PBS-1% agarose pads for imaging.

### Imaging of mixed colonies.

Fluorescence images of colonies were acquired following growth using a Nikon Eclipse Ti-E inverted fluorescence microscope equipped with a DU897 electron-multiplying charge-coupled device (EMCCD) camera (Andor) using μManager v. 1.4 (52) or a Nikon TE-2000 or Zeiss Axio Zoom.V16 microscope. Colonies sandwiched between two agar surfaces and the colonies in the corresponding control experiments were imaged after 7 days at 4°C. Colonies that did not show sufficient fluorescence at this point were imaged again after 26 more days at 4°C.

Edges of colonies were imaged with a 20X objective on a Nikon Eclipse Ti-E inverted fluorescence microscope equipped with a DU897 camera (Andor) using μManager v. 1.4.

### Identifying colony radius and sector boundaries in mixed colonies.

Images were imported into Matlab. The difference between YFP and CFP images was used to identify boundaries of intensity using the “edge” function with the “log” method. The center of the colony was defined by fitting a circle to 20 points manually selected from the images either from the border of the colony if the colonies were small enough to fit in the image or from the border of the mixed-fluorescence sector at the center of the colony. The coordinates of the boundaries were transformed to polar coordinates based on the center of the colony. Points were mapped to traces by connecting nearest neighbors. Points too close to the center of the colony were discarded. Boundary traces were cleaned up manually; some were separated at a manually selected location when the traces clearly represented two sides of a sector, and some were removed because they clearly captured a feature that was not a sector boundary. Traces in polar coordinates were then smoothed. Some parameters (closeness to the center or the threshold for the edge detected) were modified depending on the quality of the image. All traces from all colonies of a given experiment were then pooled. The mean was obtained by bootstrapping; random traces (selected with replacement) were averaged to calculate the slope, and then the slope was integrated to obtain a mean trace of the polar angle, ϕ(*r*), starting from an initial radius, *r_i_*. This process was repeated 200 times to compute the final mean trace and the standard deviation over the 200 iterations.

All code is available in a GitHub repository at https://github.com/aarandad/ColonyChirality-KCH.

### Growth measurements in liquid culture.

OD measurements were taken with an Epoch 2 plate reader (BioTek) at 37°C with continuous shaking, and the OD at 600 nm (OD_600_) was measured at 7.5-min intervals. The maximal growth rate was calculated as the maximal slope of ln(OD) with respect to time (calculated from a linear regression of a sliding window of 11 time points) using custom Matlab (MathWorks) code.

### Single-cell imaging.

Cultures were grown overnight at 37°C in LB and diluted 1:200 into fresh medium (with antibiotics when appropriate). For phase-contrast imaging, cells were spotted onto a 1% (wt/vol) agarose pad with LB. Cells treated with A22 were exposed to the drug for 2 to 3 h before being imaged.

Phase-contrast and epifluorescence images were acquired with a Nikon Ti-E inverted microscope (Nikon Instruments) using a 100X (numerical aperture [NA], 1.40) oil immersion objective and a Neo 5.5 scientific complementary metal oxide semiconductor (sCMOS) camera or a DU885 EMCCD (Andor Technology). The microscope was outfitted with an active-control environmental chamber for temperature regulation (Haison Technology, Taipei, Taiwan). Images were acquired using μManager v. 1.4 (52). Cell contours were automatically segmented using Morphometrics (53), and a local coordinate system was defined based on the meshing algorithm from MicrobeTracker (54). Some images of cells sampled from the edge of colonies (Fig. 6) had clusters of cells that could not be segmented by Morphometrics. These images were processed with the neural network-based machine-learning framework DeepCell (55) prior to segmentation with Morphometrics.

### Twist-n-TIRF measurements.

Fluorescent WGA was added to liquid cultures to a final concentration of 25 μg/ml. After 2.5 h of growth, cells were pelleted at 8,000 × *g* for 1 min and washed with PBS once before being spotted onto 1% (wt/vol) EZ-RDM–0.2% glucose or LB agarose pads with 10 μg/ml cephalexin. For anaerobic Twist-n-TIRF experiments, cultures were grown, washed, and spotted onto agarose pads inside an anaerobic chamber. Mounted pads were fully sealed with VALAP (an equal-weight mixture of Vaseline, lanolin, and paraffin) before removal from the anaerobic chamber, and imaging was conducted immediately.

### TIRF microscopy.

Twisting measurements were performed on a Ti-E inverted microscope (Nikon, NY, USA) with a 100X objective (NA, 1.40). A 488-nm Sapphire optically pumped semiconductor laser (OPSL; Coherent, CA, USA) was coupled with a TIRF illuminator (Nikon) attached to the microscope stand. Images were acquired with a DU885 EMCCD camera (Andor, CT, USA), and synchronization between components was achieved using μManager (52).

### Cell-twisting analysis.

In Twist-n-TIRF experiments, cell contours were computationally extracted from the phase-contrast images using Morphometrics (53). The integrated WGA fluorescence enclosed within the cell contour was quantified. These values were normalized to the prebleached level and plotted as a function of the change in length, Δ*l*, from the prebleached length. Curves were then fitted to extract the slope, λ, representing the rate of fluorescence recovery due to twisting. The curves were fit over the first 3 μm of elongation to avoid noise from photobleaching after large amounts of exposure.
