# Supplementary figures and images for: Bacterial Filamentation Drives Colony Chirality
Source: mBio. 2021 Nov 2;12(6):e01542-21. doi: 10.1128/mBio.01542-21 (PMC8561393; doi:10.1128/mBio.01542-21)

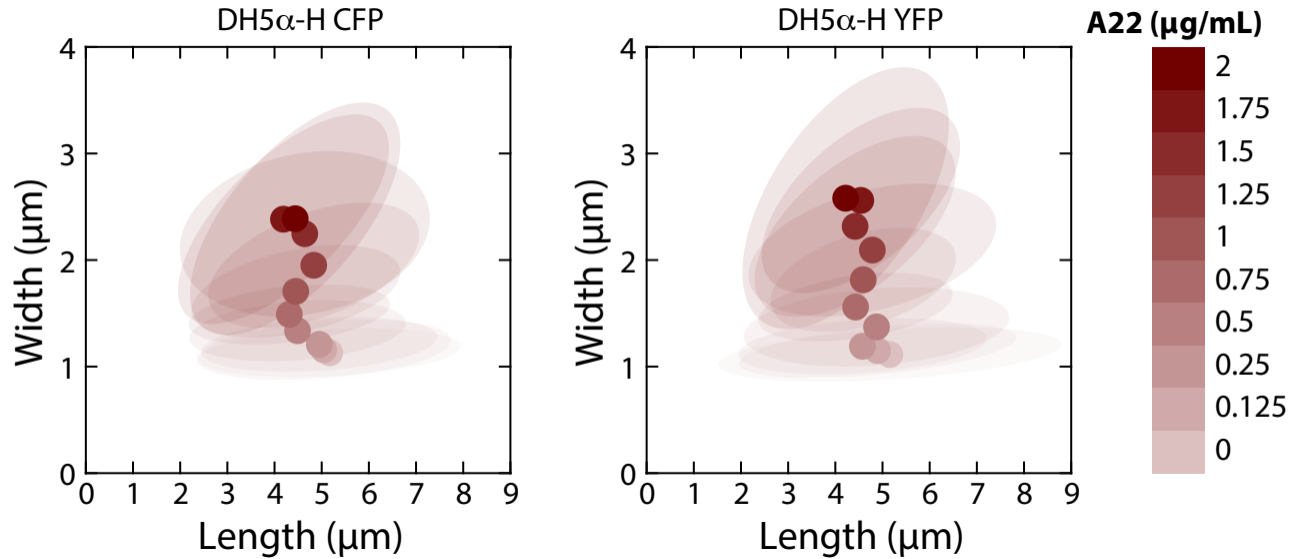

Supplement: FIG S1 [file mbio.01542-21-sf001.pdf]

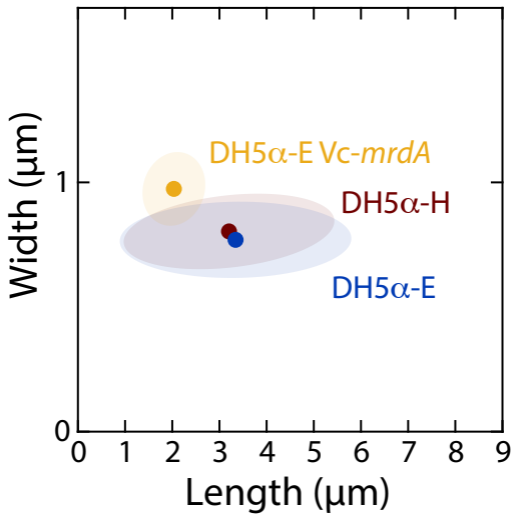

Supplement: FIG S2 [file mbio.01542-21-sf002.pdf]

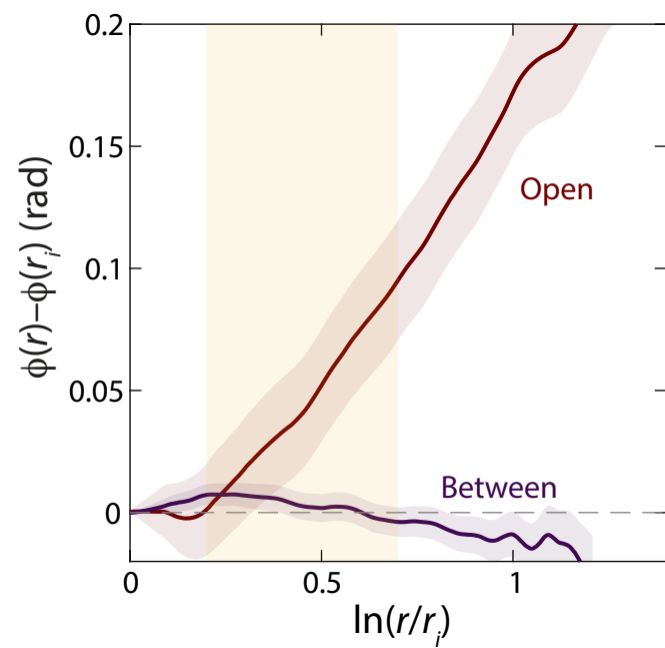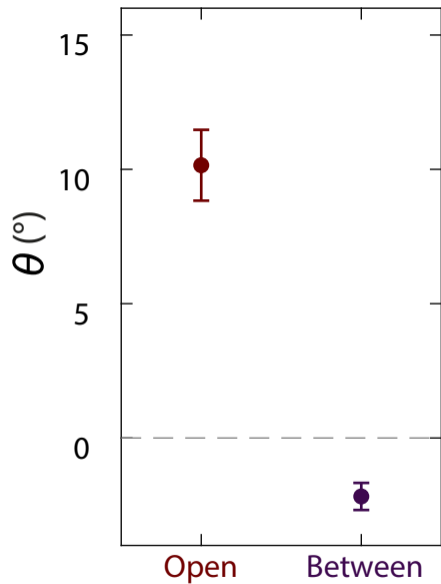

Supplement: FIG S3 [file mbio.01542-21-sf003.pdf]

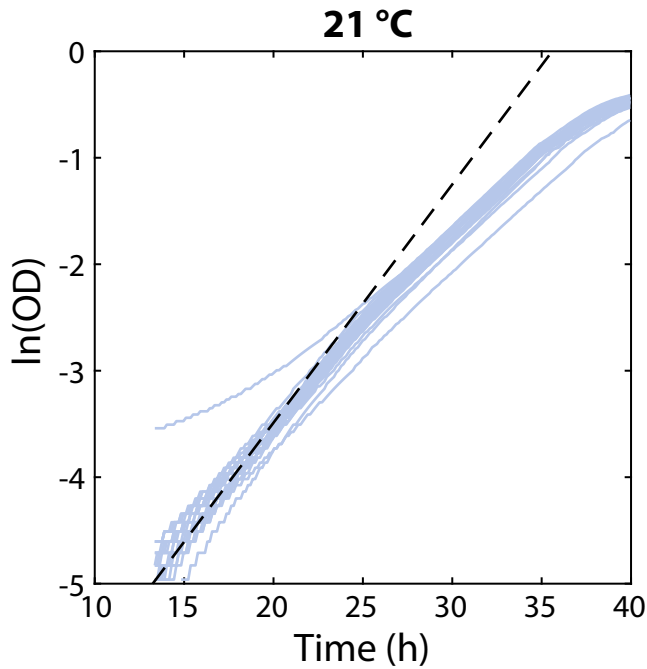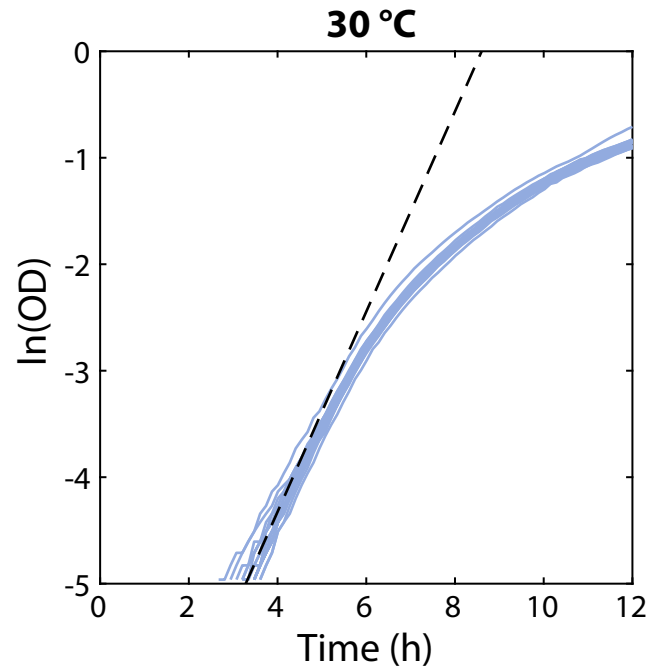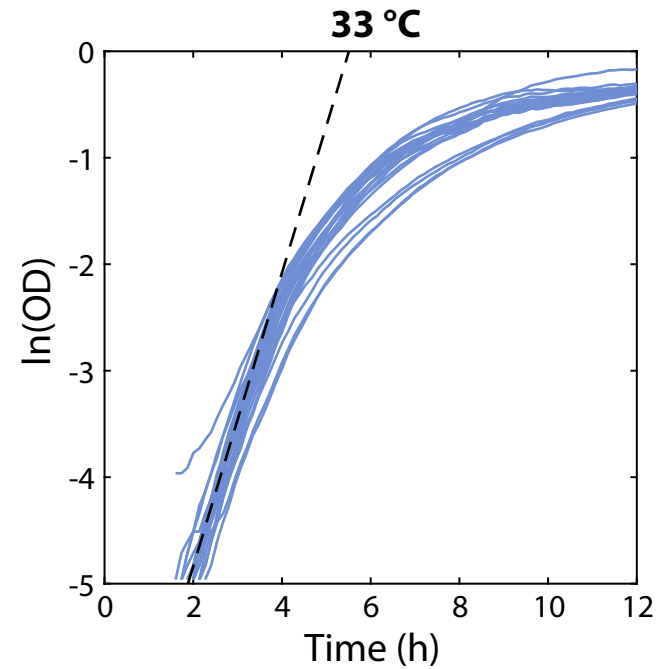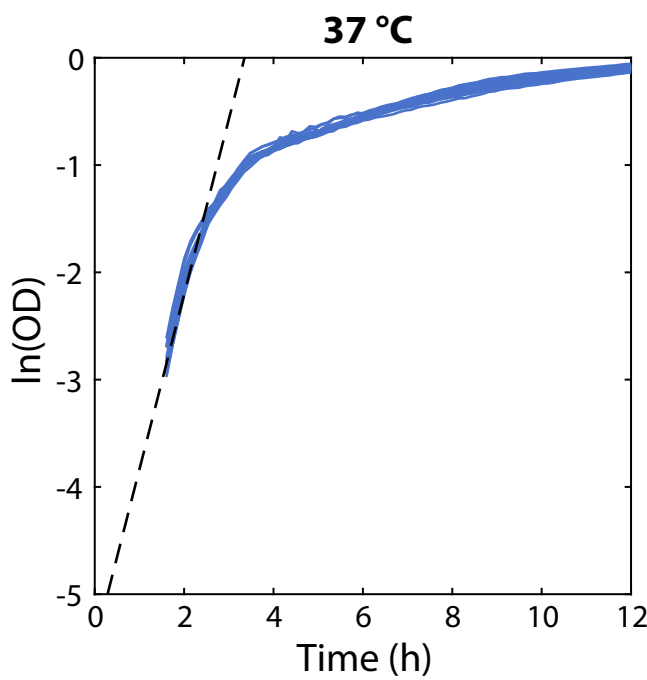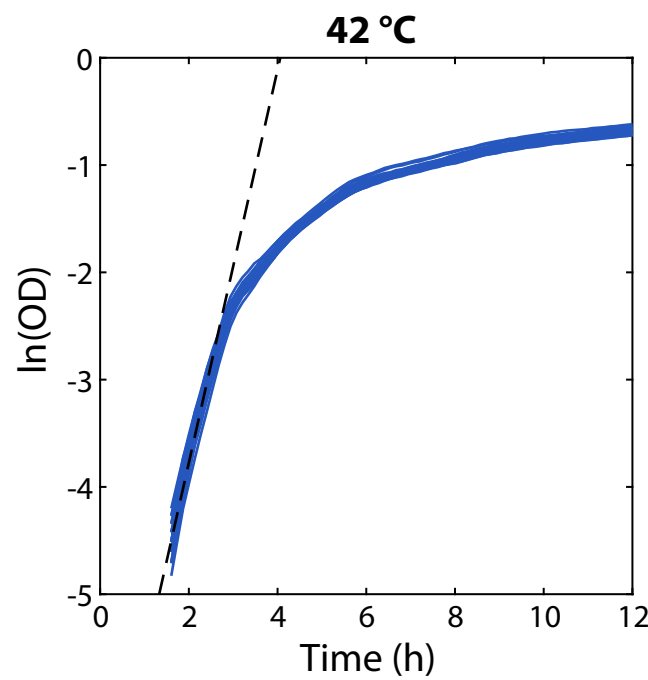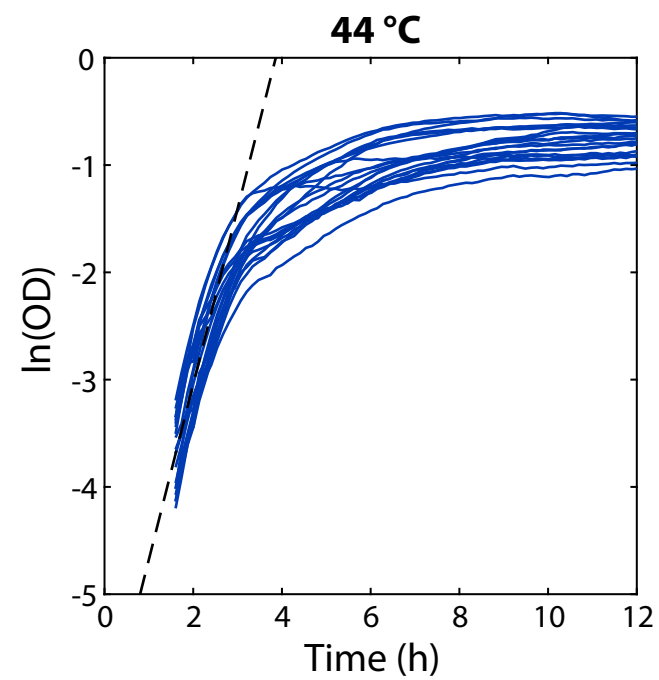

Supplement: FIG S4 [file mbio.01542-21-sf004.pdf]

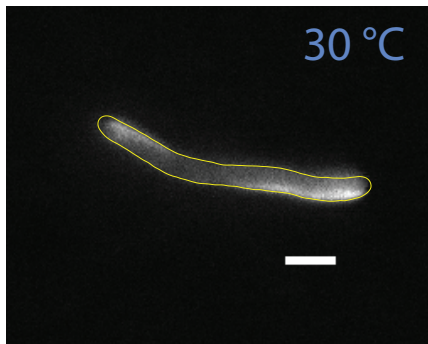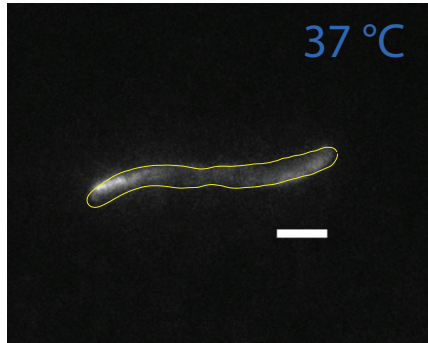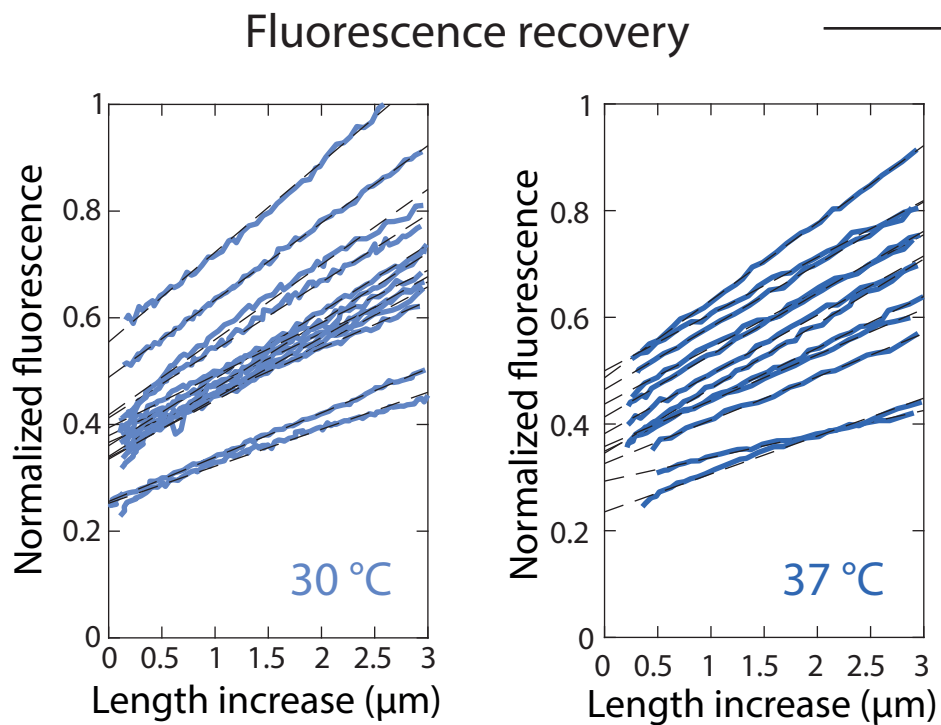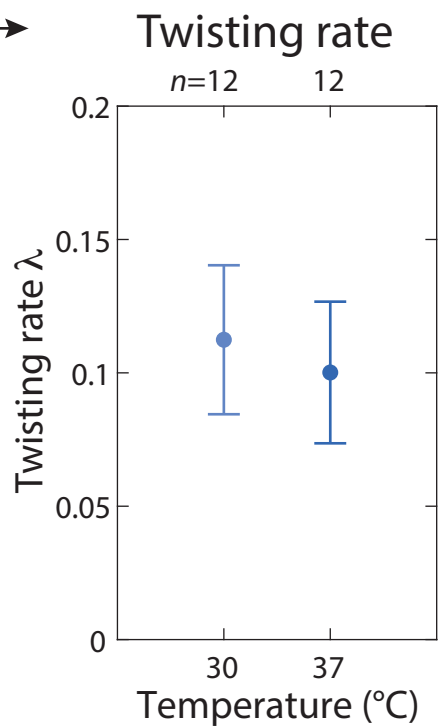

Supplement: FIG S5 [file mbio.01542-21-sf005.pdf]

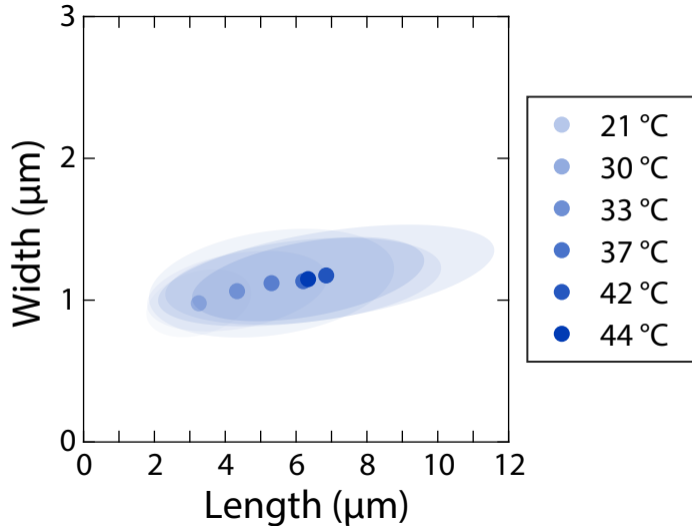

Supplement: FIG S6 [file mbio.01542-21-sf006.pdf]

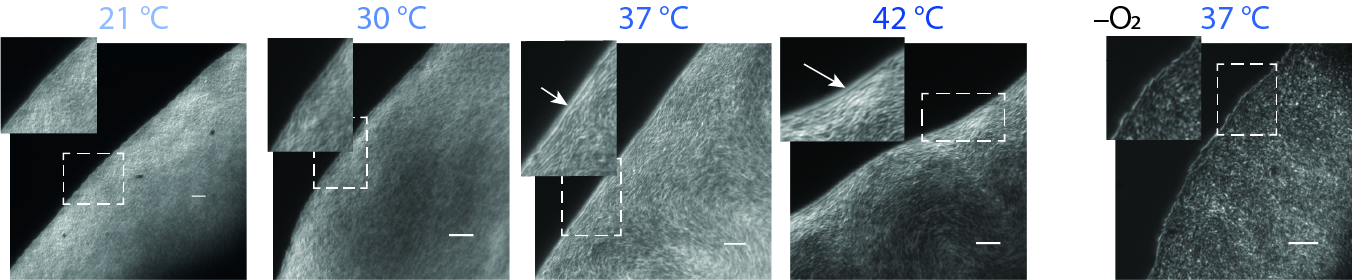

Supplement: FIG S7 [file mbio.01542-21-sf007.tif]

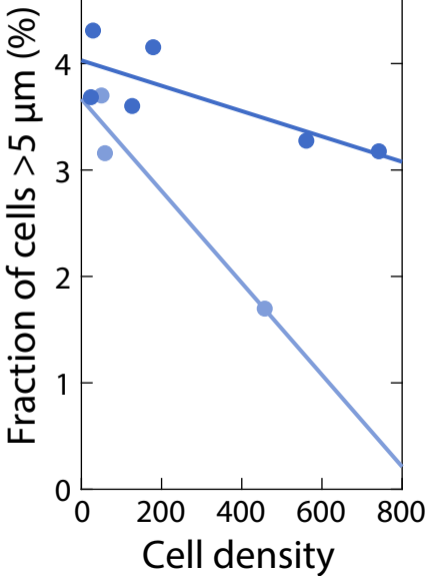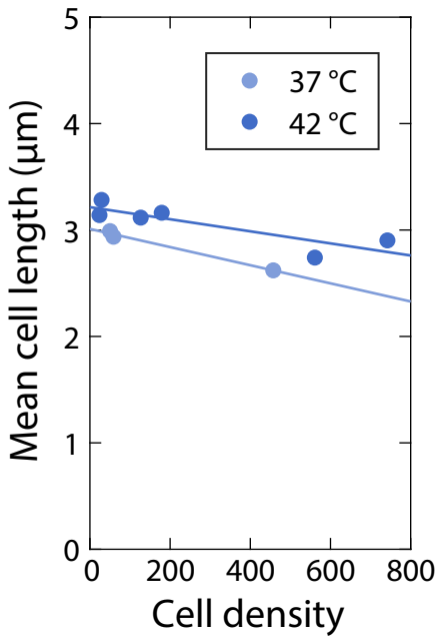

Supplement: FIG S8 [file mbio.01542-21-sf008.pdf]

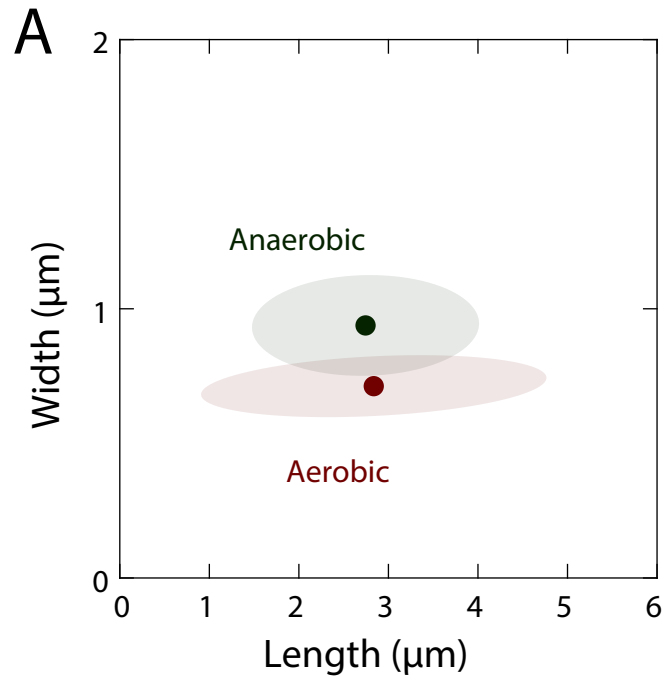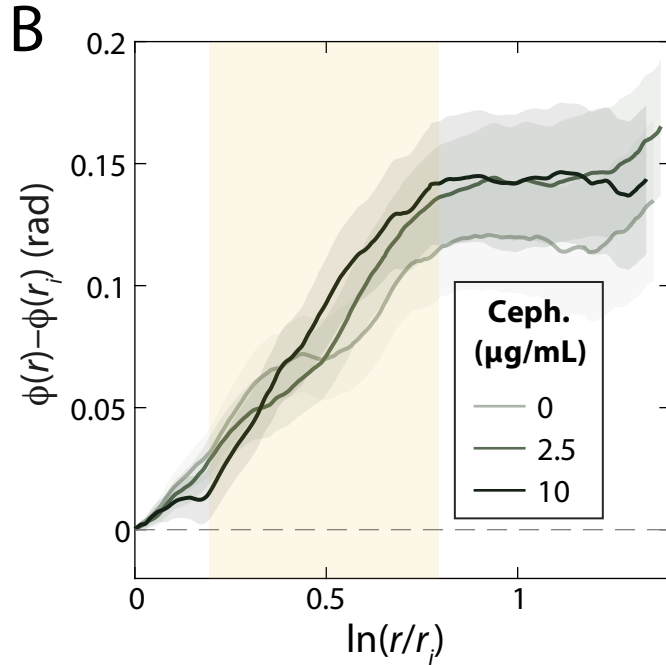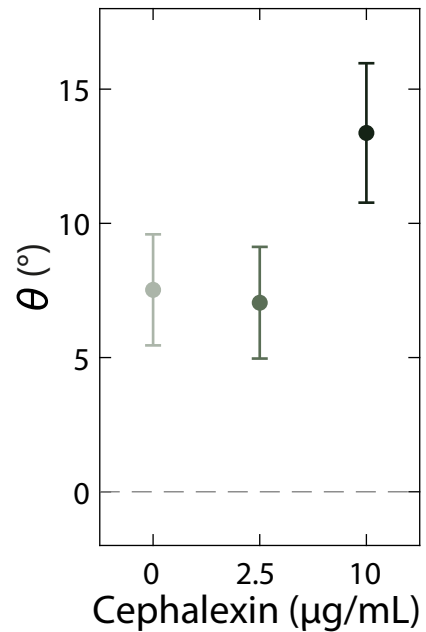

Supplement: FIG S9 [file mbio.01542-21-sf009.pdf]
