# Supplementary material for: Bacterial Filamentation Drives Colony Chirality
Source: mBio. 2021 Nov 2;12(6):e01542-21. doi: 10.1128/mBio.01542-21 (PMC8561393; doi:10.1128/mBio.01542-21)
Supplement: TABLE S1 [file mbio.01542-21-st001.pdf]

**Strain name**	**Relevant characteristics**	**Reference/origin**
DH5α-H-CFP	Plasmid pTrc99A with gene coding for CFP	(17)
DH5α-H-YFP	Plasmid pTrc99A with gene coding for YFP	(17)
DH5α-E-CFP	Plasmid pSTV28 with gene coding for CFP	This study
DH5α-E-YFP	Plasmid pSTV28 with gene coding for YFP	This study
DH5α-E-CFP Vc-*mrdA*	Plasmid as above, ΔEc-*mrdA*	This study
DH5α-E-YFP Vc-*mrdA*	Plasmid as above, ΔEc-*mrdA*	This study
